# Attitude towards mental help-seeking, motivation, and economic resources in connection with positive, negative, and general psychopathological symptoms of schizophrenia: a pilot study of a psychoeducation program

**DOI:** 10.3389/fpsyt.2024.1353125

**Published:** 2024-03-14

**Authors:** Qasir Abbas, Khawar Bilal Baig, Urooj Sadiq, Hina Ayaz Habib, Sumayah Aljhani, Zoobia Ramzan

**Affiliations:** ^1^ Department of Applied Psychology, Government College University, Faisalabad, Punjab, Pakistan; ^2^ Department of Professional Psychology, Bahria University Lahore Campus, Lahore, Pakistan; ^3^ Independent Researcher, Karachi, Pakistan; ^4^ Department of Psychiatry, College of Medicine, Qassim University, Buraydah, Saudi Arabia; ^5^ Dow International Medical College, Dow University of Health Sciences, Karachi, Pakistan

**Keywords:** positive-negative symptoms, general psychopathological symptoms, motivations, help-seeking attitude, psychoeducation program, schizophrenia disorder

## Abstract

**Introduction:**

Schizophrenia is typically treated with medication as the first approach, but additional strategies are necessary to enhance the effectiveness of this treatment for better outcomes. However, it is crucial to explore methods, alongside medication, that promote a positive attitude towards seeking mental health support and alleviate symptom severity among non-institutionalized individuals of different age groups in Pakistan. Thus, this pilot study aimed to utilize a psychoeducation program to enhance patients’ motivation and attitudes toward seeking treatment, decrease symptom severity, and investigate the role of financial factors in their illness journey.

**Methods:**

In this preliminary investigation, our focus was on individuals who had been diagnosed with schizophrenia and were receiving treatment from various hospitals and primary care clinics. Following a thorough screening process, 255 participants met the eligibility criteria, and 220 completed the psychoeducation program. The study included both male and female participants, with 143 (56.08%) being men and 112 (43.82%) being women. Regarding marital status, 123 (48.24%) were single, 98 (38.43%) were married, and 34 (13.33%) were divorced widowers or widows. The age range of the respondents varied from 18 to 52 years, with a mean age of 35.45 and a standard deviation of 10.27.

**Results:**

The results indicated a decrease in symptom severity following a 16-week psychoeducation program. The psychoeducation program significantly reduced the positive symptoms, negative symptoms, and general psychopathological symptoms among patients. Similarly, significant improvement was observed in patients’ motivation toward treatment and they actively participated in treatment after getting psychoeducation about the treatment. Similarly, after the psychoeducation program significant improvement was seen in patients’ attitudes towards help-seeking and perceived mental health functioning.

**Conclusion:**

In summary, the findings suggest that our psychoeducation program has the potential to positively impact the motivation and help-seeking attitudes of schizophrenia patients towards treatment. Moreover, there is a need for further exploration of psychoeducation programs for schizophrenia, particularly in countries facing economic challenges. This study paves the way for the development of an indigenous psychoeducation program tailored to Pakistani schizophrenia patients, with potential applicability for Urdu-speaking individuals.

**Clinical Trial Registration:**

https://www.thaiclinicaltrials.org/show/TCTR20210208003, identifier TCTR20210208003.

## Introduction

Schizophrenia is a common psychiatric condition with a wide range of behavioral and cognitive manifestations (1). Positive and negative symptoms are the main features that cause various functional impairments (2). Poor/absent insight is another major problem among patients with schizophrenia which enhances symptom severity (3, 4) and reduces patients’ motivation and help-seeking attitude toward treatment (5). Delusions, hallucinations, and disorganized speech are the usually considered more severe symptoms which usually create complicated condition (6). These symptoms cause impairment in social, behavioral, cognitive and executive functions (7). Negative symptoms causes emotional disturbance and significantly promote social and behavioral deficits (8). Overtime functional impairment changes into violence, family disputes, and illogical behavior (9). The length of disorder negatively affects patients’ views and treatment motivation and severity of symptoms also affects the therapeutic relationship, the course of treatment, and adherence (10). In contrast the patients who have positive attitude and motivations toward treatment they more likely to benefit from medicine and psychotherapy (11). Additionally, as therapy advances, individuals with a positive treatment attitude eventually feel-good emotions and become more tolerant of drug side effects (12).

Schizophrenia is worldwide common disorder. Approximately, 1 in 300 (0.32%) or 24 million individuals are suffering with schizophrenia disorder and this ratio is 1 in 222(0.45%) among adults which is very alarming (13). This incidence rate 0.3% to 0.7% is reported in The Diagnostic and Statistical Manual of Mental Disorders, Fifth Edition-text Revision (DSM-5-TR). In 2011, WHO reported the prevalence between age 15-35 is estimated 7 out of 1,000 and have been reported higher in Asian countries as compared to Japan, Australia, and the United States. An estimate made by National Mental Health India reported 0.5% current prevalence and 1.4% lifetime among Indian population (14). In Pakistan, the current prevalence is not known but an estimate made in 2015 reported 1 to 2% (15). The mortality among patients with schizophrenia is 2 tp 3% higher as compared to general population (16).

A notable deficiency seen in people with schizophrenia is motivational impairment (17). This lack of motivation appears as an internal unpleasant symptom and has been linked to poor psychosocial and functional outcomes, decreased treatment benefits, and non-adherence (18). According to Messinger et al. (19), the lack of motivation extends to a variety of areas, including less interest, desire, and curiosity as well as a lack of initiative in planning, pursuing, interacting, and following up. The treatment procedures are hampered by these impairments, which also affect the ability of decision-making (20). Additionally, Bentall et al. (21) reported patients’ expectations and performance regarding therapy are worse when there are motivational deficiencies. Karyotaki et al. (22) reported a decrease in treatment-related motivation leads to various negative treatment outcomes (i.e. therapeutic process, daily functioning, quality of life, cognitive and behavioral outcomes). Motivated patients are more likely to follow and understand treatment significance and they sustain treatment procedures (23), follow therapeutic process for change (24), and improve their ability to understand the issue (25).

Individuals from low-income groups are more vulnerable to developing psychotic disorders (26). They have the low privilege to access leisure sources, difficulty to fulfill basic needs, and social deprivation increases the chance of schizophrenia and this prevalence is higher among women (27). One of the studies reported that individuals with limited income resources have a greater chance of schizophrenic tendencies (28). Moreover, individuals who grow up with low parental income (29), live in combined houses (30), live in deprived areas (31), and face social inequality (32) and they are more likely to develop schizophrenia (33). Low-income individuals struggle to meet their fundamental needs, which worsens their health and makes it more difficult for them to get effective therapy (34).

It can be observed for this difference investigated by the WHO in 2009 that the United Stat invests 22.7 billion dollars every year to look after our individuals’ mental health services and Pakistan invests 0.4% million rupees to settle down mental healthcare facilities. This is one of the major reasons for treatment delay, dissatisfaction with treatment, and poor efficacy rate while treatment at an early stage significantly increases the chance of treatment efficacy (35). It is very unfortunate that majority of the patients are not receiving treatment facility. According to an observation, patients visit hospital for treatment, approximately, 50% diagnosed with schizophrenia and almost 31.3% can receive specialized health care facility and in low-income countries this condition is more worse (16).

Psychotropic medications are commonly considered the first choice of treatment for patients with schizophrenia worldwide (36). Similarly, in Pakistan, psychotropic medication is also a preferable choice of treatment but psychological treatment is lacking in this domain which is a major gap of treatment (37). To fill this gap, a psychoeducation program was designed for patients with schizophrenia disorder who were currently on psychotropic medicines to enhance the efficacy of treatment and that was the primary purpose of this current study. Psychoeducation program works on patients’ motivation, attitude toward treatment, treatment adherence, and symptom management. This program will help to improve patients’ motivation and positive attitudes toward treatment which will improve treatment efficacy and patients’ daily living functions. Our psychoeducation program especially included patients who had just started taking their medication as their primary treatment. We created a psychoeducation program that was used with all trial participants and through this strategy we aimed to find a useful, culturally acceptable approach to dealing the patients with schizophrenia we are interested in investigating how effective this program can be for these patients.

To investigate the effectiveness of this psychoeducation program, we structured the following hypotheses. Such as, 1) there would be a significant score difference between pre and post-assessment scores on positive, negative, and general psychopathological symptoms after completing the psychoeducation program, 2) there would be a significant score difference between pre and post-assessment scores on motivation after completing the psychoeducation program, 3) there would be a significant score difference between pre and post-assessment scores on help-seeking attitude after completing the psychoeducation program among patients with schizophrenia disorder

## Materials and methods

### Research design

This single-arm clinical trial using pre- and post-research design was conducted among patients diagnosed with schizophrenia disorder in the three biggest cities of Pakistan (i.e. Lahore, Faisalabad, & Karachi). Participants were recruited from different primary psychiatric settings, psychiatric clinics, and tertiary care hospitals between June 2017 and January 2022.

### Participants

In this study, 400 patients diagnosed with schizophrenia disorder were taken and they were assessed for eligibility assessment. In this process, 145(36.25%) patients were excluded due to reasons of 1) who did not meet the inclusion criteria n=130, 2) who provided incomplete information= 10 and who left due to other reasons were 5 participants. Finally, 255(63.75%) respondents met the eligibility criteria and were enrolled in the experimental group. The respondents’ age-ranged was between 18-50 years, with M ± SD=35.45 ± 0.27. In addition, one of the family members that he/she is closer to the patients was also taken in this study to fulfill the purpose of supporting and assistance in medication, and daily life activities to the patients at home.

### Inclusion and exclusion criteria

Based on the DSM-V’s inclusion and exclusion criteria, the study took on subjects who had been given a diagnosis of schizophrenia disorder. Subjects who had psychotic symptoms and had had their condition for at least 6 months qualified as participants. However, a few exclusion criteria were used; a) patients availing in-patients’ facility, b) with severe symptoms, c) patients with multiple relapses, d) duration of illness > 1 year and above, e) patients with comorbid conditions; and f) patients with injury/disability. Participants from various socioeconomic backgrounds were included without any particular educational requirements.

### Assessment and screening

The assessment and screening process was initiated from an outdoor patient’s psychiatric setting. After getting consent from the patient and his/her close family members than a psychological assessment was completed by the clinical psychologists. This process started with a) complete history taking form, b) a structured clinical interview, c) relevant assessment measures, and d) patients were diagnosed according to DSM-V diagnostic criteria for schizophrenia disorder. Participants who qualified for the diagnostic criteria of schizophrenia disorder were enrolled in experimental groups.

### Measures

Participants first filled out a demographic questionnaire and a history-taking form was completed. Details information was taken from the patients regarding the symptoms, duration illness, number of episodes, age at onset and related inform to confirm the diagnosis and associated factors to formulate treatment plan.

*The Positive and Negative Syndrome Scale (PANSS):* The PANSS is comprised to 30 items with 7 positive, 7 negative, and 16 general psychopathological symptoms to assess symptom severity (38). It covers three areas: general psychopathology, negative symptoms, and positive symptoms. Usually, the PANSS administration lasts between 40 and 50 minutes. In the current investigation, a licensed clinical psychologist administered the PANSS during a clinical interview. Reliability estimation of positive (α=0.73), negative (α=0.83) and general psychopathology subscale (α=0.79) which indicates scale has sound psychometric properties.

*The Mental Help-Seeking Attitude Scale (MHSAS):* The MHSAS was developed to patients attitude toward treatment (39). This nine-item self-report test is intended to gauge patients’ views toward therapy in terms of positive and negative outcomes. Patients’ low scores on the help-seeking attitude scale reflected that patients have a low attitude toward seeking help and that high scores showed parents have a positive attitude toward receiving treatment. The MHSAS has high internal consistency (α=0.94).

*Patients-Motivation for Treatment Checklist (PMTC):* The PMTC was prepared to evaluate patients’ level of motivation toward treatment. The checklist was comprised of 11 statements. Each statement was rated between “0” to “10” was used to rate each item. A score on each statement close to 0 suggests resistance to receiving treatment, score between 4 to 6 indicates a readiness to do so despite some reluctance, and a score of near to 10 indicates a strong desire to get treatment right away.

Socioeconomic status (SES) was assessed depending on the participants’ household income. Low-income patients were those whose family income per month was under 50,000 PKR, middle-income patients were those whose family income per month was between 50,000 and 90,000 PKR, and high-income patients were those whose family income per month was above 90,000 PKR.

### Treatment conditions

Psychotropic Medication: In the management of patients with schizophrenia disorder “first-generation” and “second-generation” antipsychotics play an important role for the successful recovery process (40, 41). Similarly to this, antipsychotic medications are frequently administered as the first line of treatment for schizophrenia in Pakistan (42). According to an estimate, 30 to 40% of patients respond poorly to medicine because they have low motivation, and do not receive psychotherapy (42). Antipsychotic medications’ primary goal is to reduce symptoms and allow people with schizophrenia to go about their daily lives (43). Another study reported psychotherapeutic interventions, rehabilitation, and awareness programs improve recovery along with medication and some without medication (44). This psychoeducation program was structured as a supportive intervention to improve patients’ recovery process and treatment adherence.

Psychoeducational program: This study’s psychoeducational program, based on the principles of cognitive behavior therapy, aims to inform, direct, inspire, and improve patients’ understanding of their situation (42). According to Yesilyaprak et al. (45), the treatment also addressed the patients’ dysfunctional beliefs, cognitive distortions, and low motivation. **Table 1** provides a summary of the psychoeducation program’s objectives. The course was broken down into 10 sessions, each of which focused on a different subject “i.e. psychoeducation, adherence training, motivation, cognitive conceptualization, insight development, stress management, socialization, skill training, lapse prevention, & relapse prevention”. These subjects were chosen from two main sources: 1) Cognitive Behavior Therapy-Basic and Beyond (46) and 2) schizophrenia psychoeducation program for individual with recent onset (47). where therapists can find specialized psychoeducational techniques for people with schizophrenia (48). Group activities were incorporated to encourage social interaction and networking among the patients, such as role-playing, table discussions, sharing life stories, and music and yoga exercises. The psychoeducational program’s second goal was to lessen the stigma and sense of isolation that people with schizophrenia often feel. The time between pre- and post-testing was roughly three months. Moreover, as our study involved an intervention (albeit in the pilot phase), we followed the CONSORT guidelines for reporting our study (49). Description of psychoeducation program sessions with particular goals and details are available (see **Table 1**). In addition, each patient’s family member/caregiver was approached to participate in this program for two individual sessions. The purpose of the caregiver involvement was to guide and instruct the family members about medication on time, spend quality time with patients, social and emotional support, understand and guide the patients (50). Moreover, therapist guided the family members about patients’ mental health conditions and train them to handle the patients during difficult conditions (51).

**Table 1:** Description of a psychoeducation program sessions with particular goals and details.

S. No	Treatment Goals	Contents
1	Psychoeducation	The aim was to provide patients with knowledge and understanding of the nature of their problems. This included discussing the underlying and hidden mechanisms that contribute to their difficulties. The psychoeducation sessions also involved exploring the stage of illness, evaluating its severity, and discussing the expected future outcomes.
2	Adherence training	The main aim was about educating patients regarding the importance of regularity in taking their medications. Interactively demonstrating the process of adherence. Demonstrating strategies to minimize and/or tolerate the side effects of medicines. Showing them how to make the schedule for taking their daily medicines.
3	Motivation and attitudes	The focus was on addressing impaired motivations and attitudes. Efforts were made to enhance motivation and cultivate a positive attitude towards treatment. The aim was to foster a positive mindset and outlook regarding the treatment process.
4	Cognitive conceptualization	Recognizing distortions and identifying negative schemas, thoughts, feelings, emotions, and inflexible and irrational beliefs.
5		Evaluating and addressing negative core beliefs and automatic thoughts by implementing the ABC-model. Interactive sessions to carry out cognitive restructuring techniques. Utilizing alternative thoughts and beliefs to challenge and modify existing thought patterns.
6	Insight	Collaborating with the patient’s existing level of insight. Facilitating the development of insight through therapeutic mechanisms. Establishing connections and fostering insight through the treatment process and therapeutic alliance.
7	Stress Management	Effectively managing and coping with stress. Addressing and managing worries. Providing self-help training to reduce stress. Helping individuals differentiate between healthy and unhealthy stress.
8	Socialization	Assisting and guiding the client to actively participate in social activities. Supporting the client in establishing healthy connections and relationships with the external world.
9	Skill training	Offering comprehensive training to the client in areas where they experience discomfort or difficulty in various aspects of life.
10	Relapse prevention	Educating the client on strategies to effectively control, manage, and regulate potential triggers, situations, and incidents that may occur in the future.
The psychoeducation program conducted with the members of patients’ families		
1	Psychoeducation to family	Providing a comprehensive understanding of the patient’s illness to family members and offering guidance on how they can support the patient in difficult situations through healthy discussions, appropriate coping mechanisms, and by avoiding stigmatization.
2	Social and emotional support	Educating the family on the importance of providing social and emotional support to the patients and guiding them on how to effectively offer such support to enhance the treatment process.

### Procedure

This study proposal was approved by Government College University, Faisalabad’s Institutional Review Board (IRB) in June 2017 and the study was completed in January 2022. A researcher then contacted the administrators at each institution to learn more about the settings and participant availability. The enrollment process was initially started by a researcher stationed in each participating hospital’s Out-Patient Department (OPD) by evaluating and screening patients. As previously described, individual clinical interviews and standardized tests were used to select subjects. The researcher reassured the patients of anonymity at the first session and highlighted their right to leave the study whenever they choose. A diagnostic interview and a series of self-report tests, as detailed in the measures section, were both components of the initial screening procedure. The DSM-V criteria were used to diagnose the participants, and depending on their presenting issues, they were subsequently assigned to the relevant therapies. Over the course of ten therapy sessions, participants received both medication and psychoeducation programs; in addition, two psychoeducational sessions were held with their families. The same battery of self-report measures was used for post-assessment after a period of 16 weeks.

### Evaluation of treatment acceptability

Upon completing the 16-week psychoeducation program, participants were asked to share your experience and feedback on the checklist. The purpose of this checklist was collecting patients’ feedback and their treatment experience with the therapist. each statement was rated from one (very poor) to five (very good), and participants were asked to rate each statement. The results indicated that 71% participants reported this program remain helpful for us to overcome psychological disturbance. 65% participants rated the quality of the sessions was very good, 84% expressed satisfaction with the session timing and frequency, and 59% reported we easily understand the session concept and content. 73% participants reported therapist’s behavior and handling during sessions were adequate and respecting. Almost 94% participants acknowledged, therapist properly maintained the privacy and confidentiality throughout the program. Similarly, 73% patients expressed that the involvement of family members in the treatment was good and 83% patients expressed satisfaction with the overall layout of the program.

### Statistical analysis

Using G-Power software (version 3.1.9.7), the sample size for this investigation was calculated. According to Faul et al. (52), an *a priori* effect size of 0.50, an error probability of 0.001, and a power of 0.99 led to a target sample size calculation of 138 participants using G-Power. However, 220 people in total participated in our study. Descriptive statistics (M, SD) was used to examine the sample’s demographic makeup. We used frequency distribution statistics to look at the severity of the symptoms as determined by PANSS. In order to evaluate the variations in participants’ pre- and post-test scores on the PANSS, MHSAS, and PMTC, a Wilcoxon’s Signed-Ranked test was used. We used significance level of p <0.05 and all analyses were made using IBM-SPSS version 24.

## Results

### Sample characteristics at baseline

A total 255 participants were assigned to the intervention. 20 out of 235 participants were excluded due to the following reasons (i.e. 15 patients did not complete the pre-assessment, 3 participants admitted into the in-patients setting for extensive treatment care, and 2 patients shifted to another city). At the follow-up stage 15 participants were also excluded from the analysis due to the reason of 5 participants left program after three sessions, 2 respondents move abroad with family, 3 due to relapse condition, 5 participants did not appears in post-assessment.

**Figure 1:**
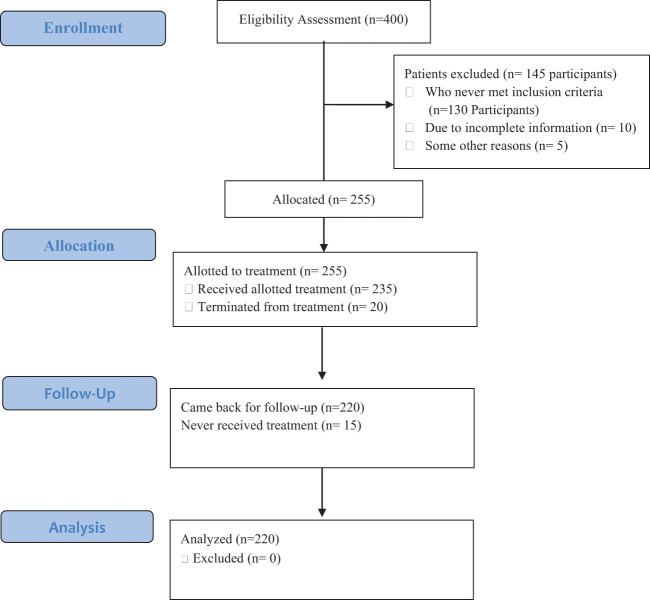
CONSORT flowchart for patients with schizophrenia disorders.

Each patient received a total of 10 therapeutic sessions. In terms of the sample characteristics, 56.08% of the participants were male and 43.82% were female. Among the participants, 48.24% were single, 38.43% were married, and 13.33% were divorced or widowed. The age of the patients ranged from 18 to 52 years, M ± SD= 35.45 ± 10.27 years. **Table 1** is list program goals and **Table 2** cover the patients’ demographic characteristics and the patients’ CONSORT flowchart presents patents’ information from enrollment to analysis (**Figure 1**).

**Table 2:** Sample demographics and baselines characteristics (n=255).

Variables	Count (%)
Gender n (%)	
Females	112 (43.82%)
Males	143 (56.08%)
Marital status n (%)	
Divorced/widow	34 (13.33%)
Single	123 (48.24%)
Married	98 (38.43%)
Education n (%)	
Master	19 (07.45%)
Bachelor	35 (13.72%)
Intermediate	65 (25.49%)
Metric	85 (33.34%)
< metric	51 (20.00%)
Family System n (%)	
Joint	121 (47.45%)
Nuclear	134 (52.55%)
Monthly Income n (%)	
90K >	85 (33.33%)
50K-90K	89 (34.90%)
< 50K	81 (31.77%)
Source of income n (%)	
Sibling support	11 (04.31%)
Labor	70 (27.45%)
Businessmen	98 (38.43%)
Employees	76 (29.81%)
Past treatment	
No treatment	22 (08.63%)
Both treatment	16 (06.28%)
Psychological (Only)	21 (08.23%)
Medication (Only)	196 (76.86%)
Duration of illness n (%)	
3 years & above	19 (07.45%)
2 years	25 (09.80%)
1year	68 (26.67%)
6-12 months	83 (32.55%)
< 6 months	60 (23.53%)
Age M (sd)	35.45 (10.27)

### Pre-interventions assessment at baseline

After being evaluated and screened, 235 volunteers who had been diagnosed with schizophrenia disorder were added to the study. The PANSS was used to assess the degree of their symptoms, and it was decided that participants with mild severity would take part in the psychoeducation program. We also concentrated on evaluating the participants’ treatment-related motives and attitudes. Participants were characterized as having a poor level of attitude toward getting help if their attitude score fell below 18, and they were categorized as having low motivation for treatment if their motivation score fell below 5 on a scale of 0–10.

The test ‘Wilcoxon signed rank’ showed a significant decrease in positive symptoms severity subsequent to our intervention. Most of the variables the effect size falls between small to medium range which reflects that the psychoeducation program was remain successful to address psychiatric symptoms in schizophrenia patients. This program brought substantial improvement in positive symptoms management i.e. delusions (51%), hallucinatory behavior (48%), suspiciousness (41%), and conceptual disorganization (40%), excitement (33%), hostility (34%) and grandiosity (29%) which shows the higher impact of the program. Program remain less effective for negative symptoms of stereotyped thinking (9%), social withdrawal and lack of spontaneity (10%) and relative better improvement was seen on blunted affect (42%), poor rapport (32%), difficulty in abstract thinking (31%) and emotional withdrawal (30%). Similarly, on general psychopathological symptoms this program significantly improves patients’ judgment and insight (53%) which was more relevant aspect to nature of the program. In addition, significant improvement was seen on depressive symptoms (49%), disturbance of volition (46%), poor impulse control and somatic concerns (42%) and on rest of the symptoms there no significant improvement was seen (see **Table 3**).

**Table 3:** Wilcoxon’s Signed-Ranked test between pre and post-testing on PANSS (N=220).

Items	Statements	Pre-Assessment M ± SD	Post-Assessment M ± SD	Z	p	r
P-7	Hostility	3.95 ± 2.29	3.43 ± 1.81	-7.04	.001	0.34
P-6	Suspiciousness	3.96 ± 2.12	3.38 ± 1.76	-8.67	.001	0.41
P-5	Grandiosity	3.66 ± 2.00	3.28 ± 1.86	-6.25	.001	0.29
P-4	Excitement	2.60 ± 1.72	2.18 ± 1.33	-6.97	.001	0.33
P-3	Hallucinatory behavior	3.75 ± 2.27	2.95 ± 1.76	-10.10	.001	0.48
P-2	Conceptual disorganization	3.14 ± 1.98	2.49 ± 1.29	-8.34	.001	0.40
P-1	Delusions	3.93 ± 2.10	3.34 ± 1.77	-10.80	.001	0.51
N-7	Stereotyped thinking	2.31 ± 0.86	2.12 ± 1.55	-1.80	.072	0.09
N-6	Lack of spontaneity	2.59 ± 1.08	2.51 ± 1.27	-2.09	.036	0.10
N-5	Difficulty in abstract thinking	2.40 ± 1.06	2.15 ± 0.95	-6.52	.001	0.31
N-4	Social withdrawal	2.27 ± 1.09	2.21 ± 1.06	-2.08	.038	0.10
N-3	Poor rapport	2.70 ± 0.84	2.50 ± 0.75	-6.63	.001	0.32
N-2	Emotional withdrawal	2.74 ± 1.11	2.45 ± 0.83	-6.31	.001	0.30
N-1	Blunted affect	2.43 ± 0.86	1.79 ± 0.93	-8.91	.001	0.42
G-16	Actual social avoidance	3.35 ± 2.06	3.31 ± 2.03	-1.43	.154	0.07
G-15	Preoccupation	2.94 ± 1.86	2.55 ± 1.46	-7.70	.001	0.37
G-14	Poor impulse control	3.08 ± 1.92	2.72 ± 1.85	-8.84	.001	0.42
G-13	Disturbance of volition	3.85 ± 1.78	3.02 ± 1.20	-9.68	.001	0.46
G-12	Lack of judgment & insight	3.03 ± 1.86	2.41 ± 1.60	-11.08	.001	0.53
G-11	Poor attention	2.04 ± 1.56	1.66 ± 1.04	-7.03	.001	0.34
G-10	Disorientation	2.58 ± 1.36	2.55 ± 1.29	-1.61	.109	0.08
G-9	Unusual thought content	2.46 ± 1.87	2.44 ± 1.82	-1.42	.157	0.07
G-8	Uncooperativeness	1.71 ± 0.88	1.56 ± 0.82	-5.83	.001	0.28
G-7	Motor retardation	1.80 ± 1.01	1.68 ± 0.92	-5.00	.001	0.24
G-6	Depression	3.06 ± 1.42	2.54 ± 1.35	-10.24	.001	0.49
G-5	Mannerism and posturing	1.96 ± 1.59	1.88 ± 1.52	-0.75	.452	0.04
G-4	Tension	2.82 ± 1.78	2.37 ± 1.30	-7.42	.001	0.35
G-3	Guilt feelings	2.90 ± 1.61	2.65 ± 1.43	-7.48	.001	0.36
G-2	Anxiety	3.10 ± 1.46	2.69 ± 1.17	-6.94	.001	0.33
G-1	Somatic concerns	2.71 ± 1.73	2.30 ± 1.49	-8.88	.001	0.42

η_p_^2^, Patrial Eta Squared; z, Wilcoxson Signed-Rank Test; ranges of r(effect size), small (effect size=0.20), medium (effect size=0.50); large (effect size ≥0.80).

The findings show that a significant difference between pre- and post-assessment scores was found after applying psychoeducation program on patients with schizophrenia disorder. For example, positive, negative and general pathological symptoms (M ± SD=21.05 ± 6.91; 15.74 ± 5.33; 38.32 ± 9.25) compared to before (M ± SD=25.00 ± 8.89; 17.44 ± 5.19; 43.40 ± 10.73) with (i.e. z =-12.47, p<.001, z=-9.52, p<.001; z =-12.72, p<.001) and i.e. r(effect size)= .59,.45 and.61 respectively. Similarly, significant improvement was found on HSAT i.e. (before M ± SD=28.27 ± 12.89) and (after M ± SD=39.03 ± 13.27) with z =-10.43, p<.001 and r(effect size)= .50. On PMFT significant improvement was observed after the implementation psychoeducation program i.e. before (M ± SD=4.85 ± 1.98) and after (M ± SD=5.69 ± 2.05) with z =-12.43, p<.001 and r(effect size)= .59 respectively (see **Table 4**).

**Table 4:** The significant difference between pre-and post-assessment on PMFT, HSAT, and PANSS (i.e. positive, negative, general symptoms) using Wilcoxon-signed Ranked Test (N=220).

Scales	Pre-assessment M ± SD	Post-assessment M ± SD	Z	p	^r^
PMFT	4.85 ± 1.98	5.69 ± 2.05	-12.43	.001	.59
HSAT	28.27 ± 12.89	39.03 ± 13.27	-10.43	.001	.50
General symptoms	43.40 ± 10.73	38.32 ± 9.25	-12.72	.001	.61
Negative symptoms	17.44 ± 5.19	15.74 ± 5.33	-9.52	.001	.45
Positive symptoms	25.00 ± 8.89	21.05 ± 6.91	-12.47	.001	.59

r, effect size, ranges of r(effect size), small (effect size=0.20), medium (effect size=0.50); large (effect size ≥0.80), HSAT, Help-Seeking Attitude Toward Treatment; PMFT, Patient’s Motivation for Treatment; PASNSS, Positive and Negative Symptoms Severity Scale.

After the implementation of the psychoeducation program patients significantly improve the positive motivation toward treatment. Findings show that on high level of motivation there 53(24.08%) participants at pre-assessment and after psychoeducation program they improve to 98(44.55%), which shows that 45(20.45%) participants improved. Similarly, 10(4.55%) improve their level of moderate motivation and almost 55(25.00%) participants improve their level of motivation from low to moderate and higher level of motivation (see **Table 5**).

**Table 5:** Change in motivation on pre- and post-assessment on PMFT after psychoeducation program with patients with schizophrenia disorder (N=220).

Scale	Pre-assessment n (%)	Post-assessment n (%)	Change n (%)
High motivation (7-10)	53 (24.08%)	98 (44.55%)	45 (20.45%)
Moderate motivation (4-6)	64 (29.10%)	74 (33.64%)	10 (4.55%)
Low motivation (<4)	103 (46.82%)	48 (21.81%)	-55 (25.00%)

Moreover, findings indicate patients with the low-income group was found significantly different as compared to patients with a middle and high-income group on positive (F= 224.70; p <. 001), negative (F= 99.71; p <.001) and general psychopathological symptoms scale (F=203.94, p <.001) among patients with schizophrenia disorder (**Table 6**).

**Table 6:** One-Way Analysis of Variance Statistics among Schizophrenia Patients on PANSS with different socioeconomic status (n=220).

Variable	Category	Descriptive Statistics			ANOVA		Tukey test (Groups Comparison)			
		*N*	*M*	*SD*	*F*	*p*	*(I)*	*(J)*	*D(I-J)*	*Sig*
*Positive Symptoms Scale (P1-P7)*										
SES	High	70	43.74	8.37			H	L	-10.07	<.000
								M	-27.23	<.000
	Middle	74	53.81	5.14	224.70	<.001	M	L	10.07	<.000
								H	-17.16	<.000
	Low	76	70.97	9.45			L	M	27.23	<.000
								H	17.16	<.000
*Negative Symptoms Scale (N1-N7)*										
SES	High	70	31.83	4.24			H	L	-12.85	<.006
								M	-24.02	<.000
	Middle	74	44.68	11.21	99.71	<.001	M	L	12.84	<.006
								H	-11.17	<.000
	Low	76	55.84	12.89			L	M	24.02	<.000
								H	11.17	<.000
*General Psychopathological Symptoms Scale (G1- G16)*										
SES	High	70	41.30	6.07			H	L	-8.70	<.000
								M	-20.66	<.000
	Middle	74	50.00	4.88	203.94	<.000	M	L	8.70	<.000
								H	-11.96	<.000
	Low	76	61.96	7.40			L	M	20.66	<.000
								H	11.96	<.000

SES, Socioeconomic Status; SS, Sum of Squares; H, High; M, Middle; L, Low.

Findings (**Table 4**) reveal that schizophrenia patients with high socioeconomic status were found significantly different as compared to patients with low and middle socioeconomic on PANSS.

**Figure 2:**
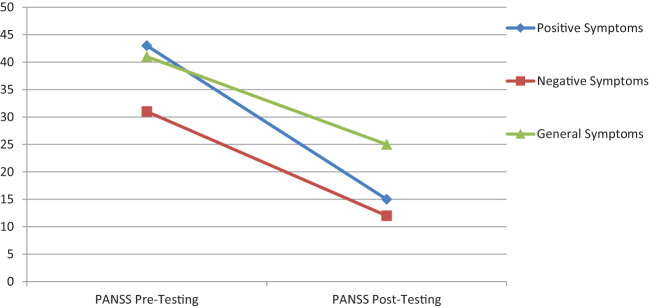
Psychoeducation program improvement from pre- to post-assessment scores on positive, negative and general symptoms severity among patients with schizophrenia disorder.

Results (**Figure 2**) show that after 16 weeks of sessions, the psychoeducation program significantly reduced symptom severity, which shows that the psychoeducational program was found an effective intervention to developing insight, understanding, motivation, and positive change in cognitive and behavioral domains.

**Figure 3:**
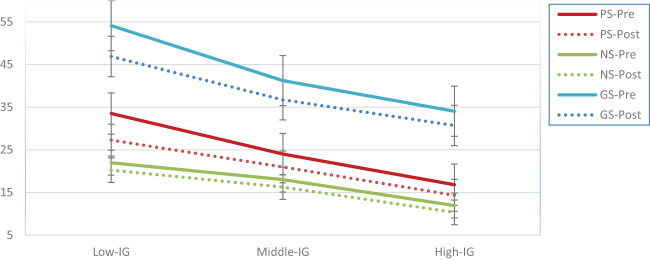
Difference in motivation level after receiving psychoeducation program among patients with schizophrenia disorder.

Findings show that the psychoeducation program significantly reduced positive, negative and general symptoms severity among all income groups while the significant improvement was observed among individuals with low income which reflects that the psychoeducation program was an effective and convenient intervention for low-income group (see **Figure 3**).

**Figure 4:**
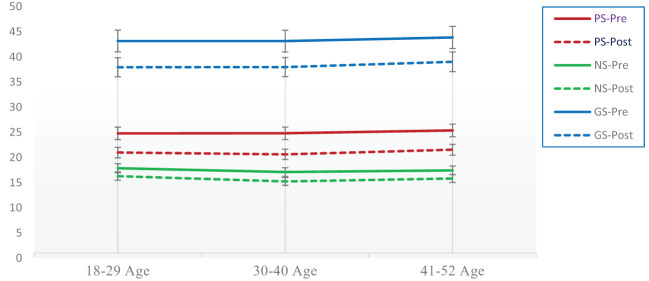
Age-wise difference between pre-and post-assessment scores on PANSS after the psychoeducation program among patients with schizophrenia disorder.

Results indicate that minor differences are seen in PANSS among patients with schizophrenia disorder after the implementation of a psychoeducation program (see **Figure 4**).

## Discussion

The current study investigates the effectiveness of a 16-week psychoeducation program with patients with schizophrenia disorder. The results showed that psychoeducation program substantially reduce the positive, negative and general psychopathological symptoms among patients. It is observed that large effect size of general psychopathological symptoms 61% and positive symptoms 59% reflects psychoeducation program remained successful to address symptoms severity. Similarly, 45% negative symptoms severity was reduced by this program These findings are consistent with the findings of the previous study, which reflects that psychoeducation program can play an important role in developing insight and reduction in symptoms severity (53). Moreover, this psychoeducation program 59% successfully improved patients’ help-seeking attitude toward treatment (54) and 50% this program remain helpful to improve patients’ level of motivation for treatment (55).

Furthermore, findings showed that this program remained successful to address positive symptoms. The effect size of positive symptoms was calculated between 30% to 51%, which showed the program significantly addressed the positive symptoms. These findings correlates with psychoeducation program conducted by Xia at al (56). Similarly, the effect size of negative symptoms indicates that this program significantly reduced blunted effect 42%, emotional withdrawal 30%, poor rapport 32% and difficulty in abstract thinking 31% but this program brought minor change in symptoms of social withdraw 10%, flow of conversation 10% and stereotype thinking 9%. These findings consistent with the past psychoeducation programs conducted among patients with schizophrenia (47, 57). This shows that the psychoeducation program is observed an effective modality to reduced negative symptoms severity. In addition, the post assessment results reported that the psychoeducation program significantly reduced the most of the general psychopathological symptoms but insignificant change was seen in mannerism, unusual thought content, disorientation and active social avoidance (58).

The usefulness of psychoeducational programs in addressing and controlling the severity of both positive and negative symptoms has been demonstrated in a number of research (50, 56, 58). Psychoeducation program promote insight and understanding which help the patients to understand the roots of the problems and patients started to change (59). Absence of psychological intervention in the treatment of schizophrenia patients is a main lacking which increases the chance of relapse and symptoms severity (60). In long term, lack of psychoeducation increases the risk of psychotic episodes, prodromal phases, and sporadic symptoms can contribute to an increase in symptom severity, eventually manifesting as negative symptoms and serious functional impairment (61–63). Psychological treatment with early onset play a significant role in symptom management (64). Our psychoeducation program enhanced patients’ behavioral and cognitive functioning which boosted their chances of recovery (65, 66)

Findings showed that our psychoeducation program played an important role in the development of motivations toward treatment (67). Our program developed an insight and understanding among patients that if they follow program instructions, they can easily overcome their emotional and behavioral problems (68). Previous evidence suggested that work on patients’ motivation toward treatment brought positive change in patients’ behavior and it is very important for chance of recovery (44). This enhanced patients’ motivation toward treatment which played an important role to address severity of positive, negative, and general psychopathological symptoms. The program gave patients the tools they needed to better control their symptoms by fostering insight and awareness. Through skill development, adherence training was essential in maintaining correct medication intake and providing patients with coping mechanisms for dealing with stressors and emotional disturbances (69).

The patients’ motivation, which is essential for their active involvement and adherence with treatment procedures, was effectively raised by this psychoeducation program. When compared to patients who have little or insufficient motivation, individuals who are sufficiently motivated tend to follow their treatment regimen more frequently. In our cultural setting, patients frequently rely only on medication without taking into account the significance of motivation and self-attitude and without being aware of the potential long-term effects. Lack of motivation is a major cause of non-compliance and the stressful cycle in which patients refuse therapy, use medications inconsistently, and have little understanding of their illness (70). High treatment motivation is essential for successful treatment outcomes and functional gains (71). Patients’ motivation was successfully increased by the psychoeducation program used in this study, encouraging optimism and active engagement in the therapeutic process (72). Internally motivated patients may strive for a higher standard of living. On the other hand, patients who are less motivated are more likely to hold unfavorable views of their therapy, think it is useless, and display more cognitive distortions (73).

Another finding reported that our psychoeducation program enhances patients help-seeking attitude toward treatment. It reflects that patients positive and help-seeking attitude toward treatment increases patients’ engagement and involvement in treatment (74). Negative attitude toward treatment profoundly affects treatment. Psychoeducational programs provided knowledge, understanding, stress management, skill development, and motivation, which shown positive change in patients’ behavior and attitudes (75). Psychoeducation program successfully encouraged adherence to treatment and improved treatment attitudes (76). Patients have been seen to have a more favorable attitude toward getting help when they feel satisfied and comply with their treatment. This psychoeducational approach produced positive results by offering direction, understanding, and therapy assistance to lessen the severity of symptoms. The program also provided therapeutic counseling and training that improved patients’ understanding of their problem, which helped them create a positive attitude about getting care. A negative help-seeking attitude toward treatment, on the other hand, might result in negative perceptions that can cause treatment avoidance and discontinuation (77).

This study found substantial differences in symptom intensity among high-income, middle-income, and low-income groups during the pretest period in addition to motivational, attitudinal, and attributional characteristics (74). However, our psychoeducation program helped all three groups, which led to a decline in symptom severity. The low-income group had the highest beginning symptom severity, but an interesting discovery was that after the psychoeducation program, their symptom severity decreased the most. The existing research frequently notes that people with schizophrenia often have worse prognoses when they originate from underprivileged families. In several nations, including India, including financial obstacles and limited access to treatment have been identified as key contributors to treatment non-compliance among patients with schizophrenia (17). Some evidence suggested that greater wealth disparity in some nations to a higher prevalence of schizophrenia (78). People with low-income group experience higher economic burden with low treatment opportunities (79) and people with more financial resources likely to benefit more from treatment (80). Additionally, patients from low socioeconomic backgrounds frequently struggle to provide for their families’ daily requirements due to a lack of access to free care or medical insurance, which further depletes their already limited resources (81). On the other hand, people with more financial resources have better access to treatment options, including better doctors and the means to pay for medicine costs, which results in lighter financial pressures (82). Additionally, the dearth of support networks and treatment facilities contributes to the symptoms getting worse over time (83, 84)

## Conclusion

The findings of this study suggest that implementing our psychoeducation program, in conjunction with medication, for the management of patients with schizophrenia, holds promise as a potential approach to reduce the frequency of positive, negative, and general psychopathological symptoms across various age groups and socioeconomic conditions. However, further confirmatory research in this area is warranted to validate these findings. The study underscores patients’ motivation and positive attitude towards seeking help as factors that could enhance engagement with treatment among schizophrenia patients. Overall, our psychoeducation program represents a way forward in the research aimed at enhancing treatment efficacy for patients with schizophrenia. However, to strengthen the evidence base, further research utilizing more robust research methods and clinical trials is necessary.

### Limitations of the study

The design of the current study has certain inherent limitations. The first thing to be aware of is that this pilot trial used a single-arm design and included people with schizophrenia who were already on medication. Because there was no control group for comparison, the efficacy of our psychoeducation program cannot be evaluated with certainty. Therefore, to establish the effectiveness of our therapy, further confirmatory trials involving a control group are required. Another limitation of the study is the psychoeducation program focuses on people with the early onset. In addition, study on emphases on symptoms severity, motivation and help-seeking which are the main factor but study does not explain the other factors such as, chronicity, comorbid conditions, in-patients treatment etc. Although, this psychoeducation program remain successful; however, it need further implementation on the factors that are not addressed in this study that would determine program further efficacy and potential. Further, research may examine the feasibility of developing this psychoeducational curriculum into a stand-alone therapy intervention. In addition, while other factors that may affect treatment outcomes were not expressly targeted, the study placed a strong emphasis on patients’ treatment motivation and attitudes. These variables include the social and emotional support networks of the patients, their cognitive abilities, their emotional quotient, their general wellbeing, and other related variables. Future studies might take into account including these components in the intervention to offer a more thorough approach to treatment. Addressing these limitations, this program may be more comprehensive in nature and it can be emerged as a evidence-based psychoeducation program for patients with schizophrenia disorder.

### The implication of the study

The findings of this psychoeducation program might be effective for therapist, practitioners and policymakers who are working with patients with schizophrenia disorders. Findings of this program promote the trends of psychotherapy and psychosocial interventions for schizophrenia patients which lacks currently in Pakistan. This pilot study sought to examine how a psychoeducation program affected schizophrenia patients’ motivation, attitudes, and symptom severity. The program’s early findings showed promising improvements in symptom severity reduction. This study’s significance comes from its ability to direct and investigate additional psychoeducation program alternatives for patients in Pakistan. For practitioners and physicians involved in the treatment of schizophrenia in Pakistan, it can be a useful resource. The study also stresses how crucial it is to start treating patients with psychotropic drugs and psychosocial assistance as well. Additionally, the results imply that psychoeducation is a powerful tool for addressing problems with treatment resistance, low motivation, and unfavorable attitudes regarding getting assistance. The motivation and attitude of patients can be addressed, which will increase therapy engagement and result in better treatment outcomes. Clinicians can modify the psychoeducation curriculum created in this study for use with other psychiatric patients who display a lack of insight and motivation for treatment. Future researchers are urged to perform comprehensive randomized control trials utilizing our program as a model using the findings of this pilot study as the basis for a useful psychoeducation program in Pakistan.

## Data availability statement

The dataset generated and/or analyzed during the present study is not publicly available because no permission was taken from the participants and the hospital administration where the study was conducted. The datasets are available from the corresponding authors upon request.

## Ethics statement

The studies involving humans were approved by Institutional Review Board (IRB), Government College University, Faisalabad, Pakistan (i.e. Ref.No.GCUF/ERC/1902, dated 05-05-2017), and then the protocol was approved by the Thai clinical trial registry (i.e. TCTR= TCTR20210208003, with URL: https://www.thaiclinicaltrials.org/show/TCTR20210208003), with first registration date 08/02/2021. Participants gave written informed consent before the process of enrollment and screening. All procedures were performed under relevant guidelines, including participant enrollment, screening, data collection, data scrutiny, and analysis. We followed the Consolidated Standards of Reporting Trials CONSORT reporting guidelines. Furthermore, we confirm that all of the procedure and methods of this study were executed by adhering to the requirements of our institutional review board, and the global code of conduct for research in resource-poor settings. The studies were conducted in accordance with the local legislation and institutional requirements. Written informed consent for participation in this study was provided by the participants' legal guardians/next of kin. Written informed consent was obtained from the individual(s), and minor(s)' legal guardian/next of kin, for the publication of any potentially identifiable images or data included in this article.

## Author contributions

QA: Formal analysis, Investigation, Supervision, Writing – original draft. KB: Conceptualization, Methodology, Validation, Writing – review & editing. US: Conceptualization, Investigation, Methodology, Resources, Writing – review & editing. HH: Formal analysis, Methodology, Resources, Software, Writing – review & editing. SA: Conceptualization, Data curation, Formal analysis, Software, Writing – review & editing. ZR: Conceptualization, Data curation, Investigation, Validation, Writing – original draft.
